# The role of adiponectin in Alzheimer’s disease: A translational review

**DOI:** 10.1016/j.jnha.2024.100166

**Published:** 2024-01-26

**Authors:** Louise Sindzingre, Elodie Bouaziz-Amar, François Mouton-Liger, Emmanuel Cognat, Julien Dumurgier, Agathe Vrillon, Claire Paquet, Matthieu Lilamand

**Affiliations:** a Université Paris Cité, UMRS 1144, INSERM, Paris, France; b Cognitive Neurology Center, AP-HP. Nord, Site Lariboisière Fernand-Widal, Paris, France; c Biochemistry Department, AP-HP. Nord, Site Lariboisière Fernand-Widal, Paris, France; d Geriatrics Department, AP-HP. Nord, Site Lariboisière Fernand-Widal, Paris, France

**Keywords:** Adiponectin, Adipokines, Metabolism, Alzheimer’s disease, Cognition

## Abstract

Adiponectin is an adipokine playing a central role in the regulation of energy homeostasis, carbohydrate and lipid metabolism, as well as immunomodulation. The relationship between Alzheimer’s disease (AD) and body composition has highlighted the bidirectional crosstalk between AD’s pathophysiology and metabolic disorders. This review aimed to report the current state of knowledge about cellular and molecular mechanisms linking adiponectin and AD, in preclinical studies. Then, we reviewed human studies to assess the relationship between adiponectin levels and AD diagnosis. We also examined the risk of incident AD regarding the participants’ baseline adiponectin level, as well as the relationship of adiponectin and cognitive decline in patients with AD.

We conducted a systematic review, in accordance with the Preferred Reporting Items for Systematic Reviews and Meta-analyses reporting guideline, of studies published over the last decade on MEDLINE and Cochrane databases. Overall, we reviewed 34 original works about adiponectin in AD, including 11 preclinical studies, two both preclinical and human studies and 21 human studies. Preclinical studies brought convincing evidence for the neuroprotective role of adiponectin on several key mechanisms of AD. Human studies showed conflicting results regarding the relationship between AD and adiponectin levels, as well as regarding the cross-sectional association between cognitive function and adiponectin levels. Adiponectin did not appear as a predictor of incident AD, nor as a predictor of cognitive decline in patients with AD.

Despite solid preclinical evidence suggesting the protective role of adiponectin in AD, inconsistent results in humans supports the need for further research.

## 1 Introduction

Alzheimer’s disease (AD) is the most prevalent cause of dementia, affecting 57 million cases worldwide in 2019, with an estimated prevalence of 153 million cases in 2050 [1]. AD is characterized by specific neuropathological lesions associating beta-amyloid (Aβ) plaques and neurofibrillary tangles composed of hyperphosphorylated tau protein [2]. The amyloid overproduction is the result of the activity of β-secretase and γ-secretase, and a decline in α-secretase disintegrin and metalloproteinase 10 (ADAM10) expression [3].

A U-shaped relationship between the body mass index (BMI) of individuals and AD diagnosis, has been suggested. Accordingly, the risk of developing AD is higher both in people with mid-life overweight and obesity and in those with a very low BMI (underweight or malnutrition) later in life [[4], [5], [6], [7], [8]]. Adipose tissue is not only an energy reserve but also an endocrine organ, capable of secreting hormones called adipokines or adipocytokines. Now, more than six hundred adipokines have been described, such as adiponectin, leptin, interleukin (IL)-6 or Tumor Necrosis Factor (TNF)-α [9,10]. In human, adiponectin monomers can form different multimers: trimers or homotrimers, hexamers and high molecular weight (HMW) multimers that represent the most predominant and biologically active forms [[11], [12], [13]]. Adiponectin targets two main receptors, adiponectin receptor 1 and 2 (AdipoR1 and AdipoR2), expressed notably in liver, muscle, heart, adipose tissue, osteoblasts, pancreas, leukocytes and kidney [14]. These receptors are also expressed in the brain in the hypothalamus, hippocampus, brainstem and brain endothelial cells in rodents and humans [10]. Activation of AdipoR1 and AdipoR2 receptors can trigger various signaling pathways, such as the 5ʹadenosine monophosphate-activated protein kinase (AMPK) and Nuclear Factor-kappa B (NF-κB) pathways. The peripheral actions of adiponectin comprise the regulation of glucose and lipid metabolism, as well as the modulation of inflammation, which both play a substantial role in AD pathophysiology.

The incidence of AD could be delayed or prevented, as several key modifiable risk factors have been pointed out [15]. Among these factors, nutritional interventions represent promising perspectives, as far as they involve complex biological pathways related to the brain function [16]. As body composition and metabolism may represent major determinants of AD pathophysiology, the role of adipokines which regulate energy expenditure, appetite or insulin resistance deserves further attention. Accumulating evidence has suggested that AD lesions may result from both impaired insulin signaling and glucose metabolism in the brain, leading to an insulin-resistant brain state [17,18]. Adiponectin is known to improve insulin sensitivity [19]. At the molecular level, the membrane insulin receptor exhibits a tyrosine-kinase activity which leads, first, to its autophosphorylation and then to tyrosine phosphorylation of insulin receptor substrates (IRS) proteins. Insulin resistance results from abnormal phosphorylation of serine/threonine residues on the receptor and/or IRS proteins. In the PI3K/Akt signaling pathway, one of the major mediator effects of insulin, Akt and GSK3β require phosphorylation at their serine residues (Akt^Ser473^ and GSK3β^Ser9^), which are reduced in insulin resistance. Furthermore, Glycogen synthase kinase 3β (GSK3β) in its phosphorylated form at tyrosine 216 (GSK3β^Tyr216^) is also involved in the excessive phosphorylation of tau. The inhibitory phosphorylation of GSK3β at serine 9 (GSK3β^Ser9^) induced by PI3K/Akt signaling pathway suppresses the hyperphosphorylation of tau [20].

The exact role and precise mechanisms linking adiponectin and AD pathophysiology are not yet clearly established. The objective of this work was to provide an updated literature review to summarize the current knowledge on the relationship between adiponectin and AD, including both preclinical and human studies. We conducted a systematic review of the available evidence to describe the cellular and molecular mechanisms linking adiponectin and AD pathophysiology, both *in vitro* and in animal models. In human studies, we aimed to assess the relationship between adiponectin levels and AD diagnosis. We also examined the risk of incident AD regarding the baseline adiponectin level as well as the relationship of adiponectin and cognitive decline in patients with AD.

## 2 Methods

### 2.1 Literature search strategy

This systematic review was conducted in accordance with the Preferred Reporting Items for Systematic Reviews and Meta-analyses (PRISMA) reporting guideline [21]. Research questions were defined to answer the research objectives: “Is there a link between adiponectin and AD lesions?” and “What would be the molecular mechanisms involved?”

We identified all published articles between January 2013 to April 2023 on PubMed interface (Medline database) and Cochrane Library. The keywords used were the Medical Subject Heading (MeSH) terms “adipokines” or “adiponectin” and “Alzheimer disease” or “amyloid” or “tau Proteins” or “neurocognitive disorders” or “cognitive dysfunction” or “dementia”. The last search was carried out on April 30, 2023.

### 2.2 Eligibility

The inclusion criteria were as follows: experimental studies on animals or *in vitro* models, humans studies, in which adiponectin levels were measured in patients with MCI or AD; meta-analysis, systematic reviews, randomized controlled trial, clinical studies, longitudinal studies (cohort or case-control; retrospective and/or prospective), and cross-sectional studies that measured adiponectin levels in patients with AD; the analysis of the link between adiponectin and AD was one of the objectives of the study ; and studies published between January 2013 to April 2023, to limit the search to the last decade evidence.

Studies regarding other neurodegenerative diseases, studies published in other languages that English or French, reviews, letters, research regarding electrophysiology experiments or based on genomics or metabolomics approaches were excluded.

### 2.3 Selection process and data extraction

After elimination of duplicates, the titles and abstracts were screened by two independent researchers. Data extraction was performed by LS and ML, using a standardized extraction form. This tool assessed the first author and journal, year of publication, study design, population, total number of participants, mean age of participants, gender ratio, diagnostic criteria for AD, stage of cognitive impairment, prevalence of type 2 diabetes, follow-up duration (when applicable), methods used for measuring adiponectin levels and the mean plasma adiponectin levels.

The final selection of the articles to be included was based on reading the full texts according to inclusion and exclusion criteria. Reasons for exclusion were documented at each stage. All papers cited in the selected articles were also examined. Each discrepancy in data extraction was solved by discussion between the researchers.

From the final selection of human studies, we aimed to bring insights into the following issues:

- What is the relationship between plasma adiponectin levels and AD diagnosis?
- Is plasma adiponectin associated with the severity of cognitive impairment in patients with AD?
- Is there a longitudinal association between baseline adiponectin levels and:
  • Incident risk of AD in patients without dementia?
  • Cognitive decline in patients with AD?

## 3 Results

A total of 2268 articles were identified using the various search terms. Overall, 11 preclinical studies, two both preclinical and human studies and 21 human studies were included in the present review (Fig. 1 in Supplementary Material).

### 3.1 Preclinical studies

**Fig. 1:**
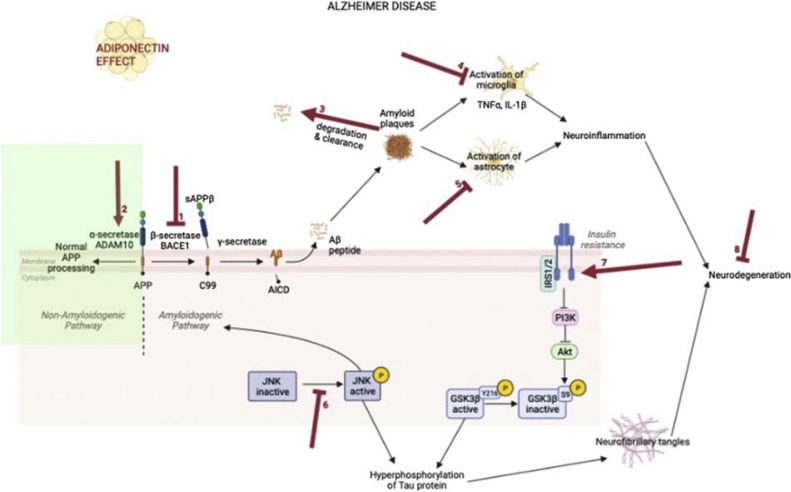
Potential effects of adiponectin in the pathogenesis of AD. APP: amyloid precursor protein; BACE1: β-site APP-cleaving enzyme-1. 1: Downregulation of BACE-1, β-secretase involve in Aβ42 formation [23,[25], [26], [27], [28]]. 2: Increase in ADAM10, involved in normal APP processing [28,30]. 3: Promotion of Aβ degradation by increasing neprilysin and clearance by autophagy activation, by enhancing lysosomal activity in microglia, by boosting the phagocytosis ability of microglia and by increasing the level of *APOE*, LDL receptor which might result into increased efflux of amyloid from the brain to plasma [22,25,29]. 4: Promotion of M2 phenotype of microglia, associated with neuroprotective roles, instead of the M1 phenotype associated with the proinflammatory response. Adiponectin would inhibit microglia-mediated neuroinflammation induced by Aβ by decreasing proinflammatory cytokines TNFα and IL-1β through AdipoR1-AMPK- NF-κB signaling pathway [24,25,29,31,32]. 5: Reduction in astrocytic activation induced by Aβ plaque deposition [[23], [24], [25]]. 6: Reduction in JNK phosphorylation, that resulted in a reduction of hyper-phosphorylation of tau protein and amyloidogenic processing of APP [25]. 7: Decreased insulin resistance *via* AdipoR1/AMPK signaling. More specifically, adiponectin could activate the PI3K/Akt/GSK-3β pathway, leading to the inhibitory phosphorylation of GSK-3β at Ser9 and thus suppressing the hyperphosphorylation of tau [[23], [24], [25],^27^,^32^,^34^,^35^]. 8: Preventing neurodegeneration induced by Aβ plaque deposition. Adiponectin would suppress neuronal apoptosis *via* p53-mediated caspase-associated apoptotic pathways. Adiponectin would promote the Aβ-induced impaired hippocampal neural stem cell proliferation through AdipoR1/AMPK/ CREB pathway [23,24,26,27,32].

Several preclinical studies have investigated the molecular and cellular mechanisms between adiponectin and some AD lesions. Two studies have shown that plasma adiponectin concentrations were lower in AD mouse-models (5xFAD) (displaying only Aβ pathology) than in wild-type (WT) mice [22,23]. In addition, adiponectin-knockout (APN-KO) mouse developed AD brain lesions including increased cerebral Aβ deposition, hyperphosphorylated tau proteins, microgliosis and astrogliosis, neuronal apoptosis and reduced synaptic proteins levels [24]. We present in Fig. 1 the main pathophysiological mechanisms underpinning the potential neuroprotective roles of adiponectin in AD.

#### 3.1.1 Amyloidogenic pathway

In the transgenic APP/PS1 mice, a mouse model of Alzheimer’s Disease developping ABéta amyloid burden via the activation of béta-secretase pathway, Khandelwal et al. and Liu et al. assessed the therapeutic efficacy of AdipoRon, an orally active adiponectin receptor agonist. AdipoRon downregulated β-site APP-cleaving enzyme-1 (BACE1) and reduced Aβ amyloid burden in both cortex and hippocampus of APP/PS1 mice [25,26]. In another transgenic model of AD, 5xFAD mice, Ng et al. also showed that AdipoRon treatment significantly reduced the levels of BACE1 through NF-κB signaling [23]. In addition, in another AD mouse model (C57BL/6J mice injected with intracerebral human Aβ) treated with intraperitoneal injection of a homolog of mammalian adiponectin from plant called osmotin, Aβ accumulation and BACE1 expression were attenuated [27]. In different *in vitro* cell models exposed to Aβ oligomers, osmotin-treated cells showed reduced expression of BACE1 [28]. Altogether these results suggest that adiponectin may downregulate BACE1 through NF-κB signaling.

Khandelwal et al. demonstrated that, in a transgenic APP/PS1 mice model, AdipoRon promoted Aβ degradation by increasing neprilysin and clearance by increasing the level of apolipoprotein E, leading to greater efflux of amyloid from the brain to plasma [25]. In another mouse model using crossed 5xFAD transgenic mice with APN-KO (5xFAD*APN-KO mice), He et al. found that adiponectin deficiency increased Aβ deposition. By the administration of AdipoRon, they demonstrated that adiponectin activated autophagy and lysosome activity in microglia, leading to the elimination of toxic accumulated Aβ [22]. Song et al. highlighted that adiponectin boosted the capacity of Aβ scavenging in microglia [29].

Abid et al. showed *in vitro* that osmotin treatment resulted in improvement in the expression of ADAM 10 [28]. Shah et al. demonstrated both *in vivo* and *in vitro* that osmotin enhanced the non-amyloidogenic gene ADAM10 in an AMPK/Silent information regulator T1 (AMPK/SIRT1)-dependent manner [30]. Osmotin treatment increased the levels of the neuroprotective fragment soluble amyloid precursor protein alpha (sAPP-α) in the brain tissues of APP/PS1 mice, indicating that it increased α-secretase activity.

#### 3.1.2 Neuroinflammation

Jian et al. showed that adiponectin deficiency increased microglia activation associated with upregulation of TNF-α and IL-1β in the cortex and hippocampus of 5xFAD transgenic mice [31]. Several studies reported that adiponectin suppressed microglial-mediated neuroinflammation induced by amyloid plaques and inhibited the proinflammatory cytokines TNFα and IL-1β. This anti-inflammatory effect in microglia is regulated through AdipoR1-AMPK-NF-κB signaling pathway [[23], [24], [25],^31^,^32^]. Moreover, several studies have shown the protective effect of adiponectin on the inflammasome, the main inflammatory mechanism in AD, *via* the AMPK pathway [33].

Song et al. showed that adiponectin promoted the induction of the microglia phenotype associated with neuroprotective roles [29]. APN-KO mice developed AD pathologies including astrogliosis [24]. AdipoRon treatment in male APP/PS1 mice or in 5xFAD and in 5xFAD*APN-KO mice reduced activation of astrocytes [23,25]. As a result, adiponectin may reduce the activation of astrocytes induced by brain Aβ deposition.

#### 3.1.3 Tau hyperphosphorylation

Several articles have shown that adiponectin may modulate the hyperphosphorylation of tau specifically through two kinases that directly phosphorylate tau: c-Jun-terminal kinase (JNK) and Glycogen synthase kinase 3β (GSK3β). Indeed, Khandelwal et al. suggested that a significant reduction in the level of hyperphosphorylation of tau in AdipoRon treated APP/PS1 mice could be due to reduced phosphorylation of JNK [25]. Similarly, with GSK3β, adiponectin promotes inhibitory phosphorylation of GSK3β at serine 9 (GSK3β^Ser9^) and thus suppressing the hyperphosphorylation of tau [[23], [24], [25],^27^,^32^,^34^,^35^].

#### 3.1.4 Insulin resistance

Aged APN-KO mice also developed hippocampal insulin resistance with increased abnormal phosphorylation of IRS level and reduced Akt^Ser473^ induction upon intracerebral insulin injection. Thus, adiponectin deficiency might lead to neuronal insulin resistance through the inhibition of the IRS signaling cascade [24]. Kim et al. reported that AD-like pathologies including insulin signaling dysfunction were highly exhibited in AdipoR1 knockdown mice, using gene therapy-mediated suppression with shRNA, consistent with brain pathologies in APN-KO mice [32]. Furthermore, in 5xFAD mice compared with their WT littermates, the hippocampal phosphorylation of Akt^Ser473^ and GSK3β^Ser9^ levels were reduced [23].

Ali et al. tested the therapeutic effect of an adiponectin-mimetic nonapeptide (Os-pep) in early-stage AD mouse models (APP/PS1 in amyloid β oligomer-injected) and in adiponectin-deficient mice (APN-KO mice). Os-pep is a very structurally stable peptide that interacts with the adiponectin recognition site on AdipoR1 and shows similar activity as adiponectin and osmotin. In AD models and in APN-KO mice, they observed neuronal insulin resistance characterized by increased levels of abnormal IRS phosphorylation, decreased levels of normal IRS phosphorylation and levels of Akt^Ser473^ and GSK3β^Ser9^. They showed that Os-pep treatment, by restoring normal phosphorylation levels, activates downstream insulin signaling *via* AdipoR1/AMPK signaling [34]. In the same transgenic mouse model of AD, APP/PS1 mice, Khandelwal et al. found that AdipoRon treatment increased phosphorylation of Akt^Ser473^, downstream to Akt increased inhibitory phosphorylation of GSK3β^Ser9^ and also activated AMPK pathway [25]. In 5xFAD mice, Ng et al. found AdipoRon treatment increased phosphorylation of Akt^Ser473^ and GSK3β^Ser9^ levels through AMPK activation, indicating that AdipoRon could improve insulin sensitivity [23]. In another study, Ali et al. found the same results in mouse Aβ1-42 model with the administration of osmotin. They observed that osmotin treatment prevented the hyperphosphorylation of tau through the regulation of PI3K/Akt/GSK-3β signaling in the hippocampus of mice Aβ1-42 models [27]. Rashtiani et al. reported that adiponectin treatment was able to boost insulin signaling by overexpression of IRS-1 protein in the hippocampus of adult male Wistar rats [35]. All this data suggest that adiponectin could reverse neuronal insulin resistance and activate downstream insulin signaling *via* AdipoR1/AMPK signaling.

#### 3.1.5 Neurodegeneration

Ng et al. and Kim and al. found that APN-KO mice as well as AdipoR1 knockdown mice respectively developed AD pathologies including increased neuronal apoptosis [24,32]. The *in vivo* study of Liu et al. showed that AdipoRon improved impaired neural stem cells proliferation in hippocampus dentate gyrus region in APP/PS1 transgenic mice. Inhibition of AMPK by compound C also reversed the promotive effects of AdipoRon on proliferation of neural stem cells in APP/PS1 mice, suggesting an AMPK-dependent mechanism by AdipoRon in AD *in vivo* [26]. In C57BL/6J mice treated by intracerebral human Aβ model, Ali et al. observed that osmotin treatment suppressed neuronal apoptosis *via* p53-mediated caspase-associated apoptotic pathways in the hippocampus [27]. Therefore, adiponectin may prevent neurodegeneration through AdipoR1/AMPK pathway.

### 3.2 Clinical studies

#### 3.2.1 General characteristics of the included studies

Overall, we retained 23 human studies in this review. They mostly included individuals with AD dementia, based on clinical diagnostic criteria such as the Diagnostic and Statistical Manual of Mental Disorders or the National Institute of Neurological and Communicative Disorders and Stroke and the Alzheimer’s Disease and Related Disorders Association criteria [36,37]. In only one study enrolling subjects with MCI, Aβ status was determined by cerebrospinal fluid (CSF) biomarkers and was part of the diagnostic criteria [38]. In three other studies, CSF amyloid and tau biomarkers measurements were performed but did not explicitly contribute to the diagnostic classification of the participants [[39], [40], [41]].

Plasma adiponectin levels were measured in 20 studies, CSF adiponectin levels were measured in one study and two studies measured adiponectin in both plasma and CSF. Most of the studies did not specify the molecular form of the measured adiponectin. When specified, total adiponectin was the most often measured. Serum adiponectin levels were mostly measured with enzyme-linked immunosorbent assays (ELISA) using kits from different manufacturers (n = 14). The other measurement methods were chemiluminescent enzyme-immunoassay, multiplex immunoassays and a few unspecified automated techniques.

Most studies underlined the role of gender in plasma adiponectin levels, with higher circulating concentrations in women [38,[41], [42], [43], [44], [45]].

The mean age of the patients ranged between 66 and 82 years. The sex ratios were variable from 33% to 100 % of females. The prevalence of type 2 diabetes varied between 0 and 33 % but was not mentioned in ten studies. In addition, the mean BMI ranged between 22.0 and 26.3 kg/m² in AD populations and between 22.6 and 28.7 kg/m² in healthy controls groups. In one study, the mean BMI value was 20 kg/m² in their whole sample and in four studies, BMI was not specified. Furthermore, one study stratified the population in two categories: non-obese (average BMI = 22,2 kg/m^2^) and obese (average = BMI 27 kg/m^2^).

#### 3.2.2 Association between adiponectin and AD diagnosis

Twenty-one studies examined the association between plasma or CSF adiponectin levels and AD diagnosis. The detailed characteristics are presented in Table 1. Ten studies found higher plasma adiponectin levels in patients with AD compared with healthy controls, or to subjects with MCI in two of these ten studies [40,46]. Baranowska-Bik et al. measured all molecular forms of adiponectin. They found higher levels of hexamers, HMW multimers and total adiponectin in AD patients than in controls, while the trimers form was lower [47]. Waragai et al. and Letra et al. measured adiponectin in the plasma and in the CSF of patients. CSF adiponectin levels were not correlated with plasma levels in both studies. Waragai et al. found lower CSF adiponectin levels in patients with AD than in healthy controls [39] and Letra et al. did not find any difference between individuals with AD versus MCI [40].

**Table 1:** Characteristics of studies regarding the relationship between adiponectin levels and diagnosis of AD in humans.

Study first author, year	Study design	Sample size	Population	Mean age (year)	Female (%)	Plasma (or CSF) level APN in AD *vs* controls	APN in AD *vs* controls (μg/ml)
Kaiyrlykyzy, 2022 [60]	Case-control study	248	98 AD	68	65%	↑	19.1 *vs* 6.2
			150 controls				
Hejazi, 2020 [45]	Cross-sectional study	60	34 AD	71	57%	↑	12.7 *vs* 9.7
			26 controls				
Bednarska-Makaruk, 2017 [61]	Cross-sectional study	425	89 AD	72	64%	↑	11.4 *vs* 8.2
			113 MCI				
			47 vascular dementia				
			69 mixed dementia				
			107 controls				
Khemka, 2014 [62]	Case-control study	120	60 AD	66	44%	↑	27 *vs* 7
			60 controls				
Baranowska-Bik, 2018 [47]	Cross-sectional study	98	58 AD	73	100%	↑	9.1 *vs* 7.7
			40 controls				
Kim, 2021 [41]	Cross-sectional study	496	95 AD	77	37%	↑	0.81 *vs* 0.69
			345 amnesic MCI				
			56 controls				
Waragai, 2016 [39]	Cross-sectional study	189	64 MCI	NA	NA	↑	18 *vs* 12
			63 AD			(↓ CSF APN)	
			62 controls				
Letra, 2019 [40]	Cross-sectional study	124	53 AD	74	69%	↑ *vs* MCI	9.8 *vs* 6.5 in MCI
			71 amnestic MCI			(∅ CSF APN, in MCI)	
Gilbert, 2018 [46]	Prospective cohort study	205	85 AD	81	65%	↑ *vs* MCI	17.0 *vs* 13.2 in MCI
			41 MCI				
			57 vascular/mixed dementia				
			11 other NCD				
			11 controls				
Li, 2020 [63]	Cross-sectional study	380	200 AD	68	100%	↑^a^	15.3 *vs* 9.7
			180 controls				
Ha, 2023 [64]	Cross-sectional study	171	82 AD	74	72%	∅	9 *vs* 8
			89 controls				
Vaňková, 2020 [42]	Cross-sectional study	102	37 AD	69	64%	∅^b^	Men: 8.9 *vs* 8.4
			65 controls				Women: 20.2 *vs* 12.5
Shang, 2018 [65]	Case-control study	673	266 AD	76	49%	∅	17.0 *vs* 14.9
			44 MCI				
			33 vascular dementia				
			200 ischemic stroke				
			130 controls				
Marcinnò, 2022 [58]	Case-control study	70	26 AD	67	57%	∅	14.3 *vs* 10.2
			21 FTD				
			23 controls				
Dukic, 2016 [66]	Cross-sectional study	235	70 AD	73	61%	∅	10.6 *vs* 8.8
			67 vascular dementia				
			48 MCI				
			50 controls				
Kim, 2022 [38]	Cross-sectional study	156	31 MCI Aβ–	75	33%	∅ Associated with Aβ status	6.7 in Aβ+ *vs* 6.3 in Aβ−
			125 MCI Aβ+				
He, 2021 [22]	Cross-sectional study	80	39 AD MCI	NA	NA	↓	5 *vs* 7
			41 controls				
Macesic, 2017 [67]	Cross-sectional study	143	62 AD	71	63%	↓	0.015 *vs* 0.019
			41 MCI				
			25 controls				
Teixeira, 2013 [68]	Cross-sectional study	157	41 AD	71	74%	↓	0.052 *vs* 0.063
			65 amnestic MCI				
			51 controls				
Ng, 2021 [23]	Cross-sectional study	10	5 AD	82	NA	(↓ CSF Hexameric APN)	NA
			5 controls				
Chen, 2021 [69]	Cross-sectional study	90	60 AD	72	53%	↓	5.18 *vs* 8.14
			30 controls				

AD: Alzheimer disease; APN: adiponectin; Controls: normal cognition healthy individuals; CSF: cerebrospinal fluid; FTD: frontotemporal dementia; MCI: mild cognitive impairment; NA: not available; NCD: neurocognitive disorder.

a Non-significant association between APN and AD diagnosis after multiple adjustments.

b Significant association between APN and AD diagnosis in women, but not after age and BMI adjustments.

Three studies also examined CSF biomarkers of amyloid and tau in AD patients. CSF adiponectin levels were correlated with CSF Aβ42 levels and negatively correlated with CSF phosphorylated tau (p-tau) levels and, in contrast to CSF adiponectin, serum adiponectin levels were not significantly correlated with AD biomarkers (CSF Aβ42 and CSF p-tau levels) [39]. However, in Letra et al.’s study, CSF adiponectin levels were not correlated with CSF Aβ42, total-tau (t-tau), and p-tau levels in the whole cohort. Interestingly, in female AD patients, there was a strong positive correlation between CSF adiponectin levels and CSF Aβ42 levels, which remained significant after age and BMI adjustments [40]. Finally, in Kim et al.’s study, serum adiponectin levels were positively correlated with CSF t-tau and p-tau levels and negatively correlated with CSF Aβ42 levels in AD *APOE4* non-carrier males [41].

Six studies did not find any association between plasma adiponectin levels in patients with AD compared with healthy subjects. Specifically, Kim et al.’s study compared amyloid positive versus amyloid negative patients with MCI and did not find any association between amyloid status and plasma adiponectin levels [38]. Five studies found a decreased level of adiponectin, in plasma in four and in CSF in one, in patients with AD compared with healthy subjects.

#### 3.2.3 Association between adiponectin and cognitive impairment

Eight studies examined the association between cognitive impairment in AD and adiponectin levels. Their characteristics are shown in Table 2.

**Table 2:** Characteristics of studies of the relationship between plasma adiponectin levels and cognitive impairment of AD in humans.

Study first author, year	Study design	Sample size	Population	Mean age (year)	Female	Mean MMSE in AD group	Association with cognitive impairment?
He, 2021 [22]	Cross-sectional study	80	39 AD MCI	NA	NA	NA	Lower MMSE when lower ADPN
			41 controls				
Kaiyrlykyzy, 2022 [60]	Case-control study	248	98 AD	68	65%	15	Lower MMSE when lower APN
			150 controls				
Chen, 2021 [69]	Cross-sectional study	90	60 AD	72	53%	NA	Lower MMSE when lower APN
			30 controls				
Hejazi, 2020 [45]	Cross-sectional study	60	34 AD	71	57%	12	No association with cognitive impairment
			26 controls				
Baranowska-Bik, 2018 [47]	Cross-sectional study	98	58 AD	73	100%	NA	No association with cognitive impairment
			40 controls				
Waragai, 2016 [39]	Cross-sectional study	189	63 AD	NA	NA	NA	No association with cognitive impairment
			64 MCI				
			62 controls				
Vaňková, 2020 [42]	Cross-sectional study	102	37 AD	69	64%	22	Lower MMSE when higher APN
			65 controls				
Khemka, 2014 [62]	Case-control study	120	60 AD	66	44%	10	Lower MMSE when higher APN
			60 controls				

AD: Alzheimer disease; APN: adiponectin; Controls: normal cognition healthy individuals; MCI: mild cognitive impairment; MMSE: Mini-Mental State Examination; NA: not available.

Cognitive impairment was assessed in all studies by the Mini-Mental State Examination (MMSE) score, and additionally by the Clinical Dementia Rating (CDR) scale in one of the studies [22]. The results of the eight studies were inconsistent. Thus, an association between lower MMSE score and lower plasma adiponectin level was demonstrated in three studies, an association between lower MMSE score and higher plasma adiponectin level was found in two other studies, while three studies did not find any association between cognitive performance and adiponectin levels.

Baranowska-Bik et al. who measured all molecular forms of adiponectin in plasma, found an association between lower MMSE and higher trimers form concentrations exclusively [47]. Despite the lack of association between MMSE score and plasma adiponectin level, Waragai et al. found that lower MMSE score were associated with lower CSF adiponectin levels [39].

#### 3.2.4 Association between adiponectin and risk of AD

The association between incident AD and plasma adiponectin levels at baseline was examined in three studies. Their characteristics are described in Table 3. With follow-up times ranging from 2.5 to 11 years, none of them found an association between plasma adiponectin levels and incident AD.

**Table 3:** Characteristics of studies of the relationship between adiponectin levels and risk of AD in humans.

Study first author, year	Study design	Median follow-up	Population at baseline	Population at the end of follow-up	Mean age	Female	Association with incident AD later in life
Kitagawa, 2016 [43]	Prospective cohort study	6.9 years	466 participants with normal cognition	27 AD	68 years	41%	∅ (HMW APN)
				20 other dementia			
				419 normal cognition			
Mooldijk, 2022 [44]	Case–cohort study	11 years	1122 participants with normal cognition	269 AD	74 years	57%	∅
				91 other dementia			
				762 normal cognition			
Teixeira, 2013 [68]	Prospective cohort study	2,5 years	54 MCI	19 AD	71 years	74%	∅
			43 normal cognition	45 MCI			
				33 normal cognition			

AD: Alzheimer disease APN: adiponectin; HMW: high molecular weight; MCI: mild cognitive impairment.

#### 3.2.5 Association between adiponectin and cognitive decline

Three longitudinal studies have studied the cognitive decline of patients according to baseline plasma adiponectin level. Their characteristics are shown in Table 4.

**Table 4:** Characteristics of studies of the relationship between plasma adiponectin levels and cognitive decline of AD in humans.

Study first author, year	Study design	Median follow-up	Population	Mean age (year)	Female (%)	Cognitive assessment	Association with cognitive decline?
Kim, 2021 [41]	Prospective cohort study	5,3 years	80 MCI APOE4 non-carrier males	78	0%	ADNI-MEM	No association with cognitive decline
						ADNI-EF	
Gibert, 2018 [46]	Prospective cohort study	1,2 years	85 AD	81	65%	MMSE score (Mean MMSE all population: 22)	No association with cognitive decline in all population
			41 MCI				
			57 vascular/mixed dementia				
			11 other NCD				
			11 controls				
Kim, 2022 [38]	Longitudinal study	4,5 years	31 MCI Aβ–	75	33%	ADAS-Cog score	Higher plasma adiponectin levels predicted the faster cognitive decline in Aβ+ but not in Aβ−
			125 MCI Aβ+				

AD: Alzheimer disease; MCI: mild cognitive impairment; MMSE: Mini-Mental State Examination.

ADNI-MEM is a memory composite score calculated from the items in several memory tasks, including the Rey Auditory Verbal Learning Test, ADAS-Cog, Logical memory and MMSE.

ADNI-EF is a executive composite score calculated from Category Fluency-animals, Category Fluency-vegetables, Trails A and B, Digit span backwards, Wechsler Adult Intelligence Scale-Revised Digit Symbol Substitution and 5 Clock Drawing items (circle, symbol, numbers, hands, time).

Study populations were different in term of gender ratios, stage of cognitive impairment, cognitive assessment methods, follow-up time as well as baseline adiponectin level, which makes it difficult to compare these studies. However, one of them reported that higher baseline plasma adiponectin levels predicted faster cognitive decline only in patients with MCI with an amyloid positive status, while the other two studies found no association.

## 4 Discussion

This translational review of the literature aimed to discuss preclinical evidence considering the role of adiponectin in the pathophysiology of AD as well as its measurements data, in both CSF and blood in adults with clinical AD, eventually in regard to AD biomarkers.

On the one hand, preclinical studies brought clear and consistent information supporting the protective role of adiponectin on several key mechanisms of AD, leading to the production of the core amyloid and tau lesions. Adiponectin appeared to decrease the synthesis of Aβ peptides and promote the degradation of amyloid deposition. In addition, adiponectin could decrease neuroinflammation and protect from neurodegeneration. Moreover, adiponectin may prevent the activation of kinases responsible for the abnormal hyperphosphorylation of tau through various mechanisms, including the improvement of insulin sensitivity. Adiponectin may bridge the gap between type 2 diabetes and the risk of incident AD as insulin resistance and oxidative stress in the central nervous system are key features of both diabetes and AD [15,17,48].

Adiponectin deficiency in AD models (5xFAD*APN-KO) and adiponectin-deficient mice (APN-KO) was associated with greater cognitive impairment. Furthermore, adiponectin supplementation improved cognitive functions and behavioral performances in several AD mice and adiponectin deficiency models.

On the other hand, human studies showed conflicting results regarding the relationship between AD and adiponectin levels. To be noted, most published results were difficult to interpret because of the heterogeneity of the study populations and of the methods used for adiponectin measurement. Indeed, adiponectin concentrations may be influenced by the characteristics of the study populations such as age, gender ratio, body composition or the prevalence of diabetes [49]. Ten studies showed a positive association between adiponectin levels and AD diagnosis while five studies showed a negative association. Six studies did not find any association. Several hypotheses may explain this paradoxical increase in plasma adiponectin concentration, in AD patients. Firstly, the elevated adiponectin levels may indicate an outdated compensatory mechanism. To counteract the insulin resistance and neuroinflammation of AD, adiponectin expression could, in response, increase. However, this increase would not be sufficient to slow down the progression of amyloid and tau lesions in AD. Secondly, adiponectin signaling could be impaired in the presence of AD lesions, as previously suggested for leptin [50]. Although adiponectin levels are increased, receptor signaling or activity could be impaired. Thus, the neuroprotective effects of adiponectin would be ineffective and insufficient to mitigate the development of brain lesions. This phenomenon could correspond to “adiponectin resistance”, i.e. a reduction in the cellular and tissue response to adiponectin, analogous to insulin resistance or leptin resistance in obesity and in AD [[51], [52], [53]]. Finally, this increase in adiponectin could be due to reduced adipose tissue mass and function. Decreased fat mass associated with incident AD might increase adiponectin secretion, independent from the core brain lesions. Thus, increased adiponectin levels could be a consequence of changes in body composition accompanying the disease, rather than a direct cause of AD.

Regarding the cross-sectional association between cognitive function and adiponectin, the studies reported inconsistent results: the mean MMSE score was lower when adiponectin level was lower in three studies, while two other studies reported an inverse association. Adiponectin did not appear as a predictor of incident AD nor as a predictor of cognitive decline in patients with AD. It is worth noting that only one study suggested that patients with MCI with an amyloid positive status showed faster cognitive decline when they had higher baseline adiponectin levels.

Several limitations must be acknowledged. One of them was the diagnosis criteria used to define patients with AD. None of the 23 studies used the most recent biological definition of AD, relying on the biomarkers amyloid and tau. Only one study used CSF amyloid status to stratify patients, but the levels of tauopathy were not specified [38]. To address this major concern, future studies should consider biomarkers-confirmed diagnoses of AD, in line with the last National Institute on Aging and Alzheimer’s Association Research Framework [54].

The sample sizes were not uniform and homogeneous between the different studies analyzed. Except for the Mooldijk et al. study, a case-cohort study that used a large sample of 1122 subjects, the sample sizes of the studies were relatively small, undermining the power and significance of the studies [44]. The population groups were also heterogeneous with patients with AD or other major neurocognitive disorders (frontotemporal dementia, vascular dementia, mixed dementia) being compared with cognitively intact healthy individuals or MCI subjects. Studies differed regarding mean age, gender ratios, prevalence of diabetes and stages of cognitive impairment. All these differences may explain the differences in terms of body composition and adiponectin levels, but also the differences in cognitive performance. These limitations may participate to the divergent results observed.

Adiponectin serum measurement methods were heterogeneous and the molecular forms studied were multiple. Plasma total adiponectin levels were strongly associated with plasma HMW adiponectin levels [55]. A strong positive correlation between plasma total adiponectin levels and CSF total adiponectin levels has been demonstrated in subjects without neurological disorders [56]. In contrast, a threshold effect suggests saturated transport across the blood-brain barrier as adiponectin levels in CSF did not increase with higher plasma levels.

The main strengths of this review were the stringent methodology conducted in accordance with the PRISMA reporting guideline and a comprehensive view linking the pathophysiological issues demonstrated in preclinical studies with the known human clinical data. Finally, adiponectin may be only one factor among many hormonal determinants of the pathophysiology of AD. Leptin [57], resistin [58,59], adipsin [42] and possibly other adipokines may also be associated with body composition and with AD.

## 5 Conclusion

This work has gathered convincing evidence from preclinical studies regarding a protective role of adiponectin driven by several key established mechanisms leading to the onset of AD lesions. In contrast, conflicting results in humans suggest the need for further research based on stringent characterization of patients with AD according to their amyloid and tau status to better understand the exact and timely role of adiponectin in AD. The promising therapeutic results in animals may open new perspectives for similar studies in humans.

## Funding

Not applicable.

## Conflicts of interest

The authors declare that they have no competing interests.

## Ethical standards

Not applicable.
