# Supplementary figures and images for: The role of adiponectin in Alzheimer’s disease: A translational review
Source: J Nutr Health Aging. 2024 Jan 26;28(3):100166. doi: 10.1016/j.jnha.2024.100166 (PMC12880398; doi:10.1016/j.jnha.2024.100166)

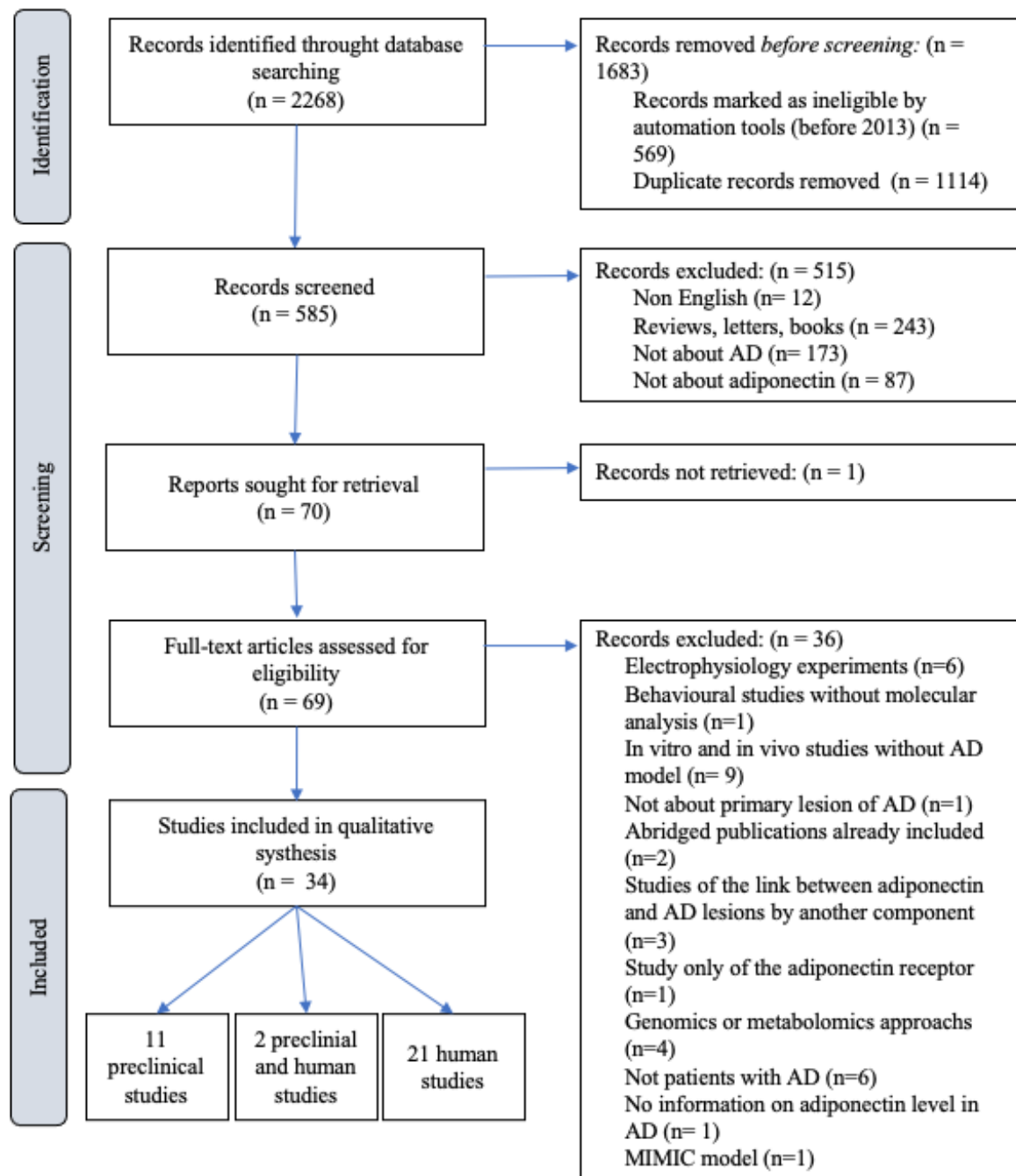

Supplement: Supplementary file 1 [file mmc1.pdf]
